# Sesquiterpene Lactone Deoxyelephantopin Isolated from *Elephantopus scaber* and Its Derivative DETD-35 Suppress BRAF^V600E^ Mutant Melanoma Lung Metastasis in Mice

**DOI:** 10.3390/ijms22063226

**Published:** 2021-03-22

**Authors:** Biljana Cvetanova, Meng-Yi Li, Chung-Chih Yang, Pei-Wen Hsiao, Yu-Chih Yang, Jia-Hua Feng, Ya-Ching Shen, Kyoko Nakagawa-Goto, Kuo-Hsiung Lee, Lie-Fen Shyur

**Affiliations:** 1 School of Pharmacy, College of Medicine, National Taiwan University, Taipei 100, Taiwan; d00423105@ntu.edu.tw (B.C.); ycshen@ntu.edu.tw (Y.-C.S.); 2 Agricultural Biotechnology Research Center, Academia Sinica, No. 128, Sec. 2, Academia Road, Nankang, Taipei 115, Taiwan; r05b22005@g.ntu.edu.tw (M.-Y.L.); yangcc@narlabs.org.tw (C.-C.Y.); pwhsiao@gate.sinica.edu.tw (P.-W.H.); ycyang12@gate.sinica.edu.tw (Y.-C.Y.); d98b42008@ntu.edu.tw (J.-H.F.); 3 Department of Biochemical Science and Technology, College of Life Science, National Taiwan University, Taipei 106, Taiwan; 4 School of Pharmaceutical Sciences, College of Medical, Pharmaceutical and Health Sciences, Kanazawa University, Kanazawa 920-1192, Japan; kngoto@p.kanazawa-u.ac.jp; 5 Division of Chemical Biology and Medicinal Chemistry, UNC Eshelman School of Pharmacy, University of North Carolina, Chapel Hill, NC 27599-7568, USA; khlee@unc.edu; 6 Chinese Medicine Research and Development Center, China Medical University and Hospital, Taichung 401, Taiwan; 7 Graduate Institute of Pharmacognosy, Taipei Medical University, Taipei 110, Taiwan; 8 Institute of BioPharmaceutical Sciences, National Sun Yat-sen University, Kaohsiung 804, Taiwan

**Keywords:** BRAF^V600E^ metastatic melanoma, deoxyelephantopin, DETD-35, oxidative stress, mitochondria dysfunction

## Abstract

Melanoma is a highly metastatic disease with an increasing rate of incidence worldwide. It is treatment refractory and has poor clinical prognosis; therefore, the development of new therapeutic agents for metastatic melanoma are urgently required. In this study, we created a lung-seeking A375LM5*^IF4g/Luc^* BRAF*^V600E^* mutant melanoma cell clone and investigated the bioefficacy of a plant sesquiterpene lactone deoxyelephantopin (DET) and its novel semi-synthetic derivative, DETD-35, in suppressing metastatic A375LM5*^IF4g/Luc^* melanoma growth in vitro and in a xenograft mouse model. DET and DETD-35 treatment inhibited A375LM5*^IF4g/Luc^* cell proliferation, and induced G_2_/M cell-cycle arrest and apoptosis. Furthermore, A375LM5*^IF4g/Luc^* exhibited clonogenic, metastatic and invasive abilities, and several A375LM5*^IF4g/Luc^* metastasis markers, *N*-cadherin, MMP_2_, vimentin and integrin α4 were significantly suppressed by treatment with either compound. Interestingly, DET- and DETD-35-induced Reactive Oxygen Species (ROS) generation and glutathione (GSH) depletion were found to be upstream events important for the in vitro activities, because exogenous GSH supplementation blunted DET and DETD-35 effects on A375LM5*^IF4g/Luc^* cells. DET and DETD-35 also induced mitochondrial DNA mutation, superoxide production, mitochondrial bioenergetics dysfunction, and mitochondrial protein deregulation. Most importantly, DET and DETD-35 inhibited lung metastasis of A375LM5*^IF4g/Luc^* in NOD/SCID mice through inhibiting pulmonary vascular permeability and melanoma cell (Mel-A+) proliferation, angiogenesis (VEGF+, CD31+) and EMT (*N*-cadherin) in the tumor microenvironment in the lungs. These findings indicate that DET and DETD-35 may be useful in the intervention of lung metastatic BRAF^V600E^ mutant melanoma.

## 1. Introduction

Melanoma, which develops from the malignant transformation of melanocytes, accounts for 2–4% of skin cancer incidence [1,2]. Even though the annual incidence of non-melanoma skin cancers is far greater (over five million in United States alone), melanoma is the most fatal skin cancer and causes the vast majority (50–75%) of all cutaneous cancer deaths [2,3]. Over the past four decades, the incidence of cutaneous melanoma has quadrupled, and melanoma is currently the fifth leading malignancy among American men following lung, prostate, colorectal and urinary bladder cancer, and the sixth leading malignancy among American women following breast, lung, colorectal, uterine corpus and thyroid cancer. The estimated number of new cases of cutaneous melanoma in situ for 2020 was around 100,000, with 6850 estimated deaths from the metastatic disease in the United States alone [4]. In melanoma patients with early diagnosis (stage I and stage II), where surgery is more curative, the five-year survival rate is 98.4%; however, in stage III, the survival rate decreases to 62.4%. For patients diagnosed with advanced melanoma (stage IV), the five-year survival rate is only 17.9% [5].

Despite its heterogeneous etiology and the highest mutation rate of any neoplastic disease, more than 50% of melanomas carry activating mutations in codon 600 of the serine–threonine protein kinase BRAF. *BRAF^V600E^* (valine to glutamine) mutation accounts for 80–90% of all the *V600* mutations in melanoma and renders increased MAPK pathway activation [6]. Although targeted therapies that center on this pathway (e.g., BRAF and MEK1/2 inhibitors) prolong patients’ survival in unresectable melanoma, most patients relapse within 6–7 months [7,8].

Melanoma is a highly immunogenic tumor, and the relevance of the immune response in melanoma was suggested half a century ago, with reports of spontaneous remission of the disease in advanced melanoma patients. These observations were supported by the increased incidence of melanoma in immunosuppressed patients, and by decreased metastasis risk in melanoma patients with increased infiltration of lymphocytes into the tumor site or the presence of melanoma-specific antibodies [9]. However, melanoma can hijack the immune system and re-educate the tumor-associated leukocytes (e.g., neutrophils, macrophages) in the tumor microenvironment to conduct an immunosuppressive role and support tumor growth and dissemination [10,11]. Remodeling of the tumor microenvironment is an emerging strategy to combat melanoma progression. Indeed, immune checkpoint inhibitors (e.g., novolumab, ipilimunab) show unprecedented efficacy in advanced melanoma patients; however, primary and acquired resistance and immune-related adverse effects are crucial obstacles to these promising immunotherapies [12,13].

Dysregulated metabolism is a hallmark of cancer cells. In addition to increased reliance on glycolysis, many cancers depend on oxidative phosphorylation (OXPHOS) for their increased demand for energy to support proliferation, invasion, and metastasis. Studies have shown that OXPHOS mediates BRAF-mutant melanoma treatment evasion [14,15]. Furthermore, the increased metabolic activities of cancer cells cause an increased production of ROS (superoxide, hydrogen peroxide, hydroxyl radical, nitric oxide, hypochlorus acid) among other intracellular by-products. Excessive levels of ROS can cause oxidative damage to macromolecules, including mutations of the mitochondrial DNA (mtDNA); therefore, cancer cells depend highly on the endogenous anti-oxidant defense system to maintain a redox homeostasis for survival [16,17]. For example, Piskounova et al. demonstrated that during metastasis, melanoma cells experience elevated oxidative stress, and that successful metastasis depends on elevated glutathione (GSH) regeneration [18]. Increased basal oxidative stress in metastatic melanoma cells suggests that pharmacological modulation of the anti-oxidant defense system or further ROS increase could be exploited to push cells over the limit and increase cancer cell selective killing [19,20]. There has been growing evidence that many phytocompounds exert their anti-cancer activities by modulating oxidative stress in cells, and several ROS-inducing phytocompounds or phytocompound derivatives are currently undergoing clinical trials [17].

Deoxyelephantopin (DET) is a germacranolide sesquiterpene lactone isolated from a medicinal plant from Taiwan, *Elephantopus scaber* L. (Asteraceae) [21,22]. Over the last decade, our lab has been extensively exploring the potential immunomodulatory and anti-cancer activity of DET in different animal models as monotherapy or in combination with clinically used therapeutics. For example, in mice, DET was shown to have a protective effect against inflammatory liver damage, a synergistic effect in combination with the chemotherapeutic drug cisplatin in B16 melanoma lung metastasis, and to attenuate cisplatin-induced nephrotoxicity and be more effective in suppressing TS/A (ER+) mammary adenocarcinoma growth and metastasis than the clinically used chemotherapeutic paclitaxel [22,23,24]. Our team used DET as a lead compound and created more potent anti-cancer molecular analog DETD-35 that showed superior suppression of MDA-MB-231 (triple negative breast cancer) cell proliferation, cell motility, migration/invasion than the parental compound and dose-dependent inhibition of MDA-MB-231 lung metastasis in mice [25]. In A375 BRAF-mutant melanoma cells, DETD-35 showed superior activity to DET in sensitizing PLX4028 (PLX) to attenuate PLX-resistant A375-R melanoma growth [26].

In this study, we created BRAF-mutant lung metastatic melanoma cells (A375LM5*^IF4g/Luc^*) and demonstrated that DETD-35 is more potent at inhibiting A375LM5*^IF4g/Luc^* activity in vitro than its parental compound DET, and that GSH depletion and ROS accumulation are important upstream events. The anti-metastatic effects of both compounds were demonstrated in vivo in a lung-seeking A375LM5*^IF4g/Luc^* melanoma xenograft mouse model. This study indicates that phyto-sesquiterpene lactone derivatives may be useful in controlling highly metastatic, late stage BRAF mutant melanoma in humans.

## 2. Results

### 2.1. Establishment of A375LM5^IF4g/Luc^ Lung-Seeking Melanoma Cells

**Figure 1:**
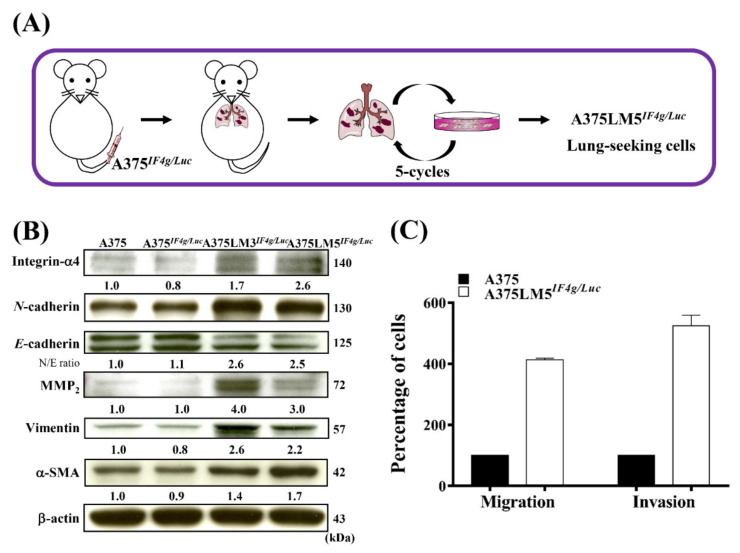
Establishment of lung-seeking A375LM5*^IF4g/Luc^* melanoma cells. (**A**) Schematic representation of the creation of lung-seeking A375LM5*^IF4g/Luc^* melanoma cells. (**B**) Western blotting was performed to check the basal levels of A375LM5*^IF4g/Luc^* metastasis-related proteins. Increased/decreased protein levels among cells are presented as a fold change to the vehicle control after normalization to β-actin. (**C**) The cell migratory and invasive ability of A375 and A375LM5*^IF4g/Luc^* were evaluated by Boyden chamber assay. The migrated/invaded cells were recorded by inverted microscopy at 10× magnification and counted in three fields. The number of migrated/invaded A375 or A375LM5 cells was first normalized to the number of seeded cells and further expressed as the migration or invasion percentage to the parental cells, where parental cell migration or invasion was designated as 100%.

To explore the anti-metastatic potential of DET and DETD-35, our team established a BRAF-mutant human melanoma lung-seeking clone that we named A375LM5*^IF4g/Luc^*. Briefly, A375 BRAF*^V600E^* human melanoma cells with stable firefly luciferase reporter genes driven by a hybrid EF1α/eIF4g promoter (named A375*^IF4g/Luc^*) were injected into the tail vein of NOD/SCID mice, and the lung metastases lesions were primarily cultured ex vivo to recover the metastatic A375LM*^IF4g/Luc^* cells. After the procedure was repeated for five cycles, A375LM1*^IF4g/Luc^*, A375LM2*^IF4g/Luc^*, A375LM3*^IF4g/Luc^*, A375LM4*^IF4g/Luc^*, and A375LM5*^IF4g/Luc^* metastatic lines were established (Figure 1A). The expression levels of proteins involved in EMT and metastasis among parental A375, A375*^IF4g/Luc^*, A375LM3*^IF4g/Luc^*, and A375LM5*^IF4g/Luc^* cell lines were examined using Western blotting (Figure 1B). Corresponding Western blotting quantification with bar graph is presented in Figure S1A. A transition from *E*-cadherin to *N*-cadherin protein expression showed that the cancer cells were in the transition phase from the benign type to the invasive, metastatic type [27]. We observed that the protein expression levels of *E*-cadherin were decreased in the A375LM3*^IF4g/Luc^* (0.7-fold) and A375LM5*^IF4g/Luc^* (0.6-fold) cells compared to the parental A375 and A375*^IF4g/Luc^* cells, whereas *N*-cadherin levels were higher in the A375LM3*^IF4g/Luc^* (1.8-fold) and A375LM5*^IF4g/Luc^* (1.5-fold) melanoma cells than the parental A375 and A375*^IF4g/Luc^* cells (Figure 1B). Matrix metalloproteinase-2 (MMP2) is involved in the breakdown of ECM and has a strong correlation to the progression of metastasis in many forms of cancer [28]. The protein expression of MMP2 in the A375LM3*^IF4g/Luc^* and A375LM5*^IF4g/Luc^* melanoma cells was higher (4.0- to 3.0-fold, respectively) than the other two parental cell lines (Figure 1B). Vimentin, an EMT marker and major component of the cytoskeleton in mesenchymal cells, also showed higher expression level in the A375LM3*^IF4g/Luc^* and A375LM5*^IF4g/Luc^* melanoma cells (2.6- and 2.2-fold, respectively) than in parental A375 and A375*^IF4g/Luc^* cell lines (Figure 1B) [28]. α-Smooth muscle actin (α-SMA), the expression of which positively influences metastatic potential, and integrin-α4, whose expression is related to enhanced metastasis, were also expressed at higher levels in both A375LM3*^IF4g/Luc^* (1.4- and 1.7-fold, respectively) and A375LM5*^IF4g/Luc^* (1.7- and 2.6-fold, respectively) melanoma cells (Figure 1B) [29,30]. These data indicate that both A375LM3*^IF4g/Luc^* and A375LM5*^IF4g/Luc^* lung-seeking cell lines indeed exhibit more metastatic potential than the A375 and A375*^IF4g/Luc^* cells. Furthermore, A375LM5*^IF4g/Luc^* had four-fold higher migratory and five-fold higher invasive ability compared to the parental A375 cells (Figure 1C). In this study, we decided to use A375LM5*^IF4g/Luc^* melanoma lung-seeking cells to examine the anti-metastatic potential of DET and DETD-35 in vitro and in vivo.

### 2.2. DET and DETD-35 Inhibited Proliferation and Induced G_2_/M-Phase Arrest in A375LM5^IF4g/Luc^ Melanoma Cells

**Figure 2:**
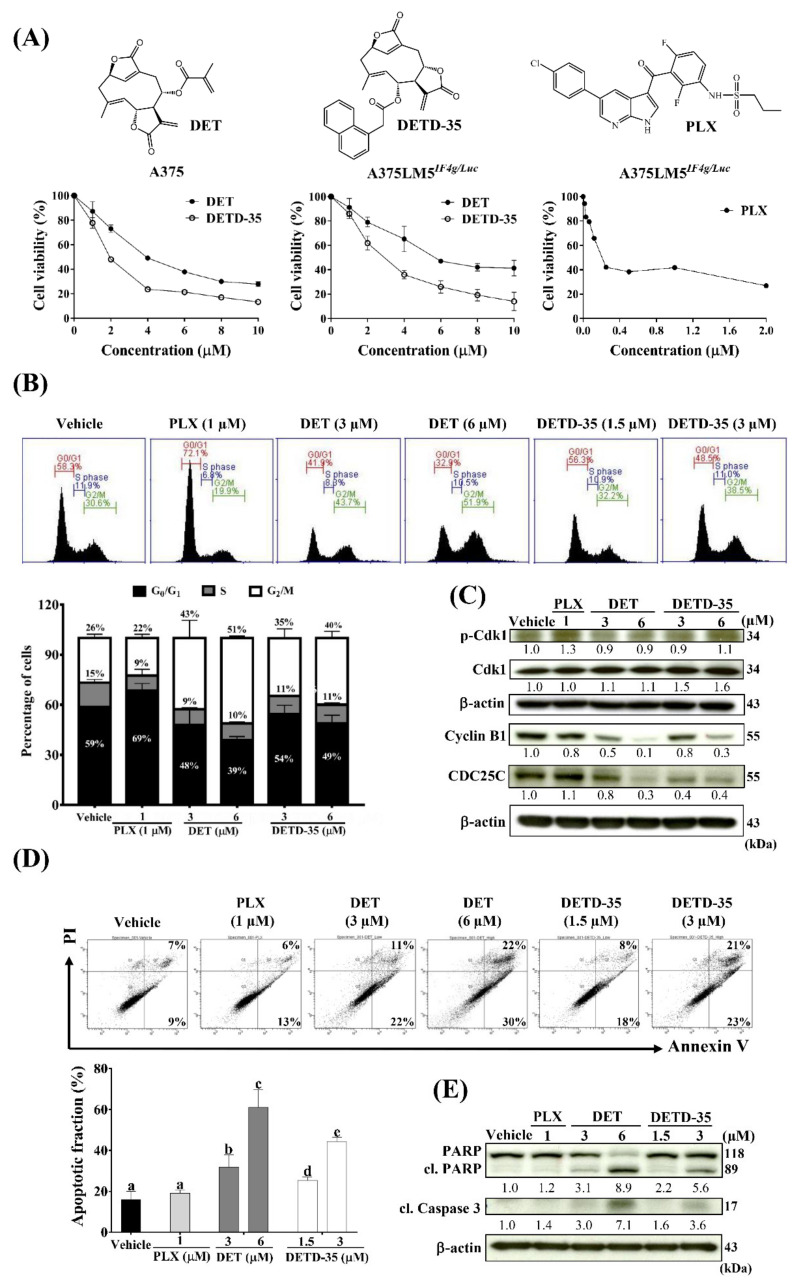
DET and DETD-35 inhibited melanoma cell proliferation and induced G_2_/M cell cycle arrest and apoptosis in A375LM5*^IF4g/Luc^* lung-seeking melanoma cells. (**A**) Cell viability was determined by MTT assay. A375 and A375LM5*^IF4g/Luc^* cells were incubated with vehicle, DET or DETD-35 for 24 h and vehicle or PLX for 72 h. (**B**) A375LM5*^IF4g/Luc^* cells were incubated with vehicle, DET, DETD-35 or PLX for 24 h. The cells were collected, fixed with ethanol, and stained with propidium iodide, and DNA distribution in the cells was analyzed by flow cytometry. Top, DNA content in the cell. Bottom, DNA percentage in G_0_/G_1_, S and G_2_/M stage. Data are mean ± SD, *n* = 3. (**C**) A375LM5*^IF4g/Luc^* cells were incubated with the same agents as in (**B**) for 24 h and the expression of the cell cycle proteins were analyzed using Western blotting. Increased/decreased protein levels among treatments are presented as a fold change to the vehicle control after normalization to β-actin. (**D**) A375LM5*^IF4g/Luc^* cells were incubated with vehicle, DET, DETD-35 or PLX for 48 h. The apoptotic population was determined by Annexin V/PI staining and flow cytometry. Top, related quadrant diagrams showing apoptotic cell status. Bottom, quantitative data are mean ± SD, *n* = 3. Means with significant differences are denoted with different letters (one-way ANOVA, *p* ≤ 0.05). (**E**) A375LM5*^IF4g/Luc^* cells were incubated with the same agents as in (**D**) for 48 h and the expression of the apoptotic proteins were analyzed by Western blotting. Increased/decreased protein levels among treatments are presented as a fold change to the vehicle control after normalization to β-actin.

DET and DETD-35 treatment for 24 h effectively inhibited A375LM5*^IF4g/Luc^* cell (5000 cells/well) viability with IC_50_ values of 5.3 µM and 2.7 µM, respectively, which was comparable to the parental A375 cells with IC_50_ values of 3.3 and 1.7 µM, respectively (Figure 2A). These data indicate that both compounds are effective in inhibiting the viability of the tested melanoma cells, with DETD-35 showing better inhibitory activity than DET. The clinical BRAF inhibitor drug PLX was used as a reference in this study, and 72 h of treatment inhibited the A375LM5*^IF4g/Luc^* cell (2000 cells/well) proliferation with a IC_50_ value of 0.21 µM (Figure 2A).

To assess the possible mechanism of growth inhibition, the effect of DET and DETD-35 on the cell cycle distribution of A375LM5*^IF4g/Luc^* cells was analyzed by flow cytometry. As shown in Figure 2B, the results revealed that upon 24 h of 3 µM and 6 µM DET treatment, 43% and 51% of the A375LM5*^IF4g/Luc^* cells underwent G_2_/M phase arrest, respectively; upon 1.5 µM and 3 µM DETD-35 treatment, 35% and 40% of the cells underwent G_2_/M phase arrest, respectively, while in the vehicle-treated control, only 26% of the cell population underwent G_2_/M phase arrest. PLX is known to induce G_1_-phase cell cycle arrest in melanoma cells, and the A375LM5*^IF4g/Luc^* cell population that underwent G_1_ -phase arrest was increased from 59% in the vehicle control to 69% upon 1 µM PLX treatment (Figure 2B). To better understand the effect of the compounds on A375LM5*^IF4g/Luc^* cell cycle machinery involved in G_2_-phase to M-phase transition, we used Western blotting to examine the protein expression pattern of some cell-cycle regulators in the treated cells (Figure 2C). Corresponding Western blotting quantification with bar graph is presented in Figure S1B. Twenty-four hours of DET and DETD-35 treatment did not show an obvious effect on p-cdk1 protein expression in A375LM5*^IF4g/Luc^* cells, while PLX treatment slightly induced p-cdk1 protein expression. The expression level of cdk1 was similar in PLX-, DET- and vehicle-treated cells, while the cdk1 protein level increased after DETD-35 treatment (Figure 2C). The expression levels of cyclin B1, important for G_2_ to M transition, was decreased dose-dependently in both DET- and DETD-35-treated cells, and a slight decrease was seen in the PLX-treated cells; similarly, CDC25C, which regulates cdk1/cyclin B1 complex, was inhibited by both compound treatments (Figure 2C). Taken together, DET and DETD-35 induced G_2_/M phase arrest in A375LM5*^IF4g/Luc^* melanoma lung-seeking cells by affecting the cdk1/cyclin B1 complex regulation and G_2_ to M transition.

### 2.3. DET and DETD-35 Induced Apoptosis in A375LM5^IF4g/Luc^ Melanoma Cells

We further examined the DET and DETD-35 treatment-induced apoptosis in A375LM5*^IF4g/Luc^* cells by PI/AnnexinV staining. Flow cytometry data demonstrated that upon 48 h treatment, the percentage of apoptotic cells increased from 16% in the vehicle group to 31% and 61% in the 3 µM and 6 µM DET groups, respectively, and to 25% and 44% in the 1.5 µM and 3 µM DETD-35 groups, respectively (Figure 2D). These results show that at the same dose of 3 µM, DETD-35 induced a higher level of apoptosis than DET (44% vs. 31%) in melanoma cells (Figure 2D). PLX induced 19% apoptosis, which was not significantly different from the vehicle group. This was expected because PLX is known to inhibit BRAF-mutant melanoma cell proliferation by inducing G_1_ cell cycle arrest with few apoptosis-related activities (Figure 2D). Furthermore, we used Western blot analysis to measure the effect of DET and DETD-35 on the expression of the most common hallmarks of apoptosis in A375LM5*^IF4g/Luc^* cells (Figure 2E). Corresponding Western blotting quantification with bar graph is presented in Figure S1C. Forty-eight hours of DET or DETD-35 treatment resulted in a dose-dependent and significant increase in the cleaved and active form of caspase 3, along with a decrease in its substrate PARP total form and an increase in cleaved PARP (Figure 2E). In contrast, PLX showed only slight or no effect on the two hallmark proteins of apoptosis. All of these results were in good agreement with the flow cytometry data mentioned above. Together, these results indicate that DET and DETD-35 inhibited A375LM5*^IF4g/Luc^* cell proliferation and viability by inducing G_2_/M arrest and apoptosis.

### 2.4. DET and DETD-35 Suppressed the Metastatic Properties of A375LM5^IF4g/Luc^ Cells

**Figure 3:**
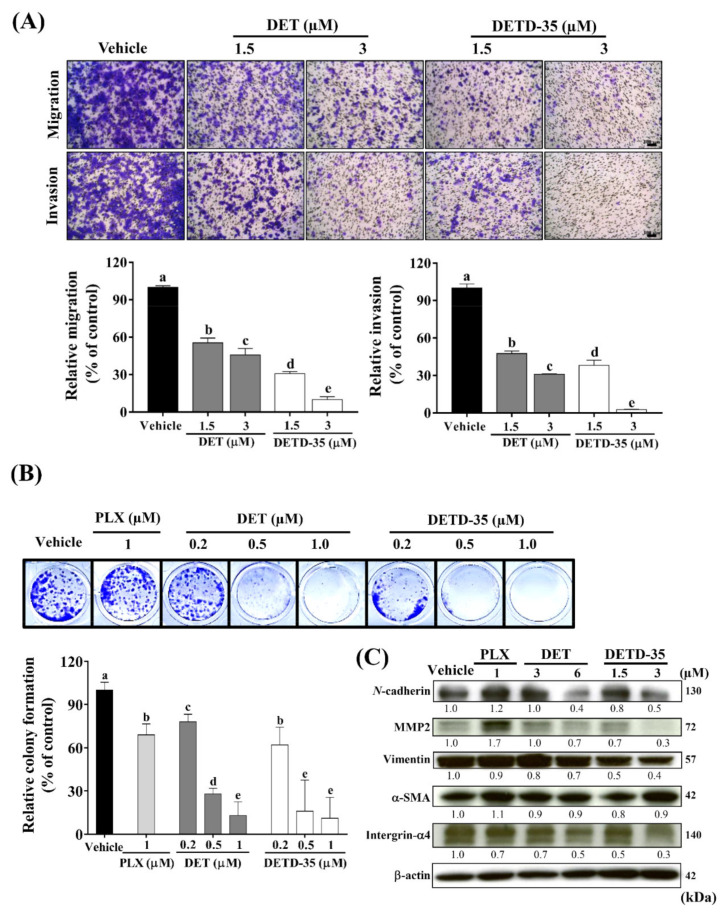
DET and DETD-35 inhibited migratory/invasive and colony forming abilities and the expression of metastasis progression markers in A375LM5*^IF4g/Luc^* cells. (**A**) The migration/invasion ability of A375LM5*^IF4g/Luc^* cells (3 × 10^4^) after DET and DETD-35 treatment were analyzed by Boyden chamber assay. Top, migrated/invaded cells stained with crystal violet. The scale bar represents 100 µm. Bottom, crystal violet intensity of migrated/invaded cells detected at OD_595_. Data are mean ± SD, *n* = 3. (**B**) A375LM5*^IF4g/Luc^* cells were incubated with vehicle, DET, DETD-35, or PLX for six days and the colony formation ability was analyzed. Top, cell colony stained with crystal violet. Bottom, quantification data of the crystal violet stained cells (OD_595_). Means with significant differences are denoted with different letters (one-way ANOVA, *p* ≤ 0.05). (**C**) A375LM5^IF4g/Luc^ cells were incubated with vehicle, DET, DETD-35, and PLX for 48 h. The expression levels of metastatic protein markers were analyzed by Western blotting. Increased/decreased protein levels among treatments are presented as a fold change to the vehicle control after normalization to β-actin.

Migration is a process of cancer cell scattering, while invasion enables the cancer cells to penetrate the basement membrane and extracellular matrix. Transwell assay was adapted to measure the migration and invasion behaviors of A375LM5*^IF4g/Luc^* melanoma cells with or without compound treatment for 12 h. DET treatment at 1.5 µM and 3 µM significantly inhibited the migratory ability of A375LM5*^IF4g/Luc^* by 45% and 54%, respectively, while DETD-35 treatment at 1.5 µM and 3 µM inhibited the cells migratory ability by 69% and 90%, respectively. The compounds had similar effects on A375LM5*^IF4g/Luc^* cell invasion ability with inhibition of 52% and 69% upon 1.5 µM and 3 µM DET treatment, and 65% and 97% upon 1.5 µM and 3 µM DETD-35 treatment (Figure 3A).

Colony formation ability enables a single cell to grow into a colony population, which is a critical step in distal organ metastasis and evasion. When the A375LM5*^IF4g/Luc^* cells were treated with either compound for 6 days at 0.2 µM, 0.5 µM, or 1.0 µM, the colony formation inhibition by DET was 22%, 72%, and 87%, respectively, and the colony formation inhibition by DETD-35 was 38%, 84%, and 89%, respectively (Figure 3B). PLX treatment at 1 µM led to 31% inhibition of A375LM5*^IF4g/Luc^* colony formation (Figure 3B). Furthermore, the effect of DET and DETD-35 on the expression of several metastasis-progression markers in A375LM5*^IF4g/Luc^* was investigated by Western blot analysis (Figure 3C). Corresponding Western blotting quantification with bar graph is presented in Figure S1D. We found that 48 h treatment with either compound resulted in a significant decrease in *N*-cadherin, MMP2, vimentin and integrin-α4, but little or no effect on α-SMA protein levels (Figure 3C). We also found that PLX treatment decreased the expression of integrin-α4, whereas it increased the levels of *N*-cadherin and MMP2 in the melanoma cells (Figure 3C). Previously it was shown that in BRAF mutant melanoma cells, PLX treatment led to a decrease in the protein levels of *N*-cadherin and MMP2 among other EMT-related markers; however, when resistance to PLX developed, there was a significant increase in the expression of these proteins [31,32]. In this study, we also found that in A375LM5*^IF4g/Luc^*, the IC_50_ of PLX was increased three-fold (Figure 2A) compared to parental A375 melanoma cells [26]. One reason for this might be that altered metastatic signaling in A375LM5*^IF4g/Luc^* drives different responses to BRAF inhibitors. These data indicate that DET and DETD-35 inhibited the metastatic ability of A375LM5*^IF4g/Luc^* cells, and at the same concentration (3 µM), DETD-35 exhibited better effects than DET.

### 2.5. ROS Scavenger GSH Reversed DET- and DETD-35-Induced Oxidative Stress-Mediated Anti-Proliferative, Apoptotic and Migratory/Invasive Effects in A375LM5^IF4g/Luc^ Cells

**Figure 4:**
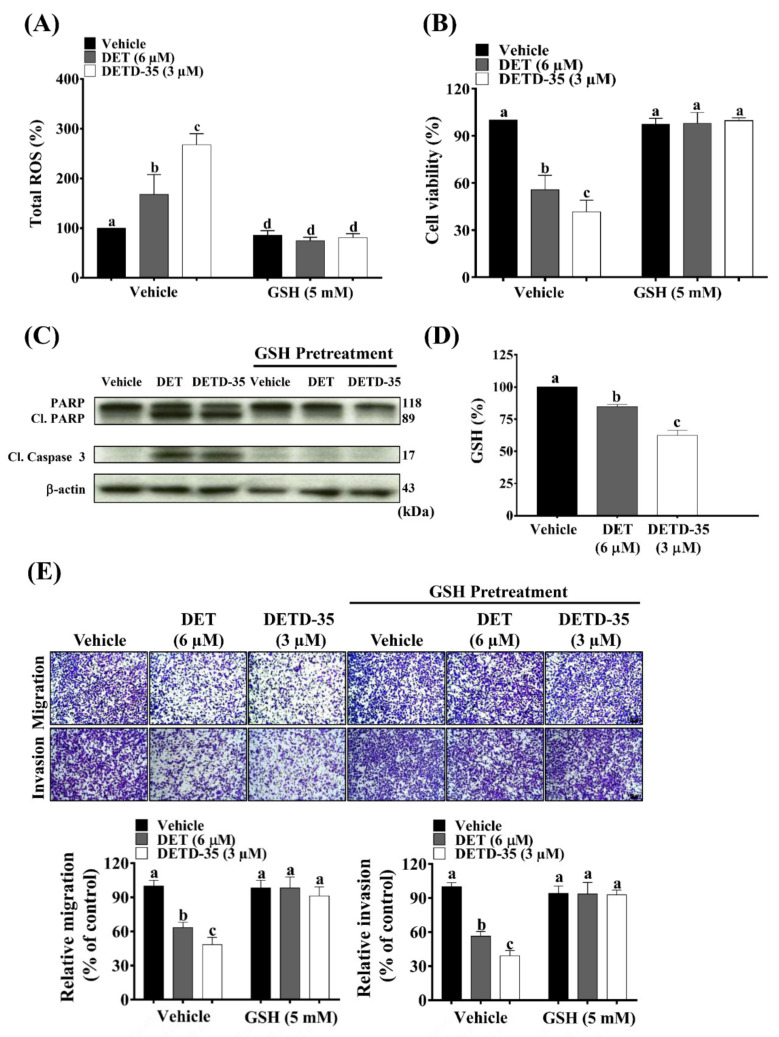
GSH reversed DET- and DETD-35-induced oxidative-stress mediated anti-proliferative and metastatic bioactivities in A375LM5*^IF4g/Luc^* cells. (**A**) A375LM5*^IF4g/Luc^* melanoma cells were pre-treated with/without ROS scavenger GSH (5 mM) for one hour and then incubated with vehicle, DET, or DETD-35 for one hour together with a green probe that can be oxidized to a green fluorescence product upon reaction with ROS. Intracellular fluorescence was measured by flow cytometry. Data are mean ± SD, *n* = 3. (**B**) A375LM5*^IF4g/Luc^* melanoma cells were pre-treated with/without GSH (5 mM) for one hour and then incubated with vehicle, DET, or DETD-35, and the cell viability was determined by MTT assay 24 h later. Data are mean ± SD, *n* = 3. (**C**) The A375LM5*^IF4g/Luc^* cells were pre-treated with/without GSH (5 mM) for one hour and then incubated with vehicle, DET (6 µM) or DETD-35 (3 µM) for 48 h, and expression levels of apoptotic protein markers were analyzed by Western blotting. (**D**) A375LM5*^IF4g/Luc^* cells were incubated with 6 µM DET or 3 µM DETD-35 for one hour and the intracellular GSH concentration was determined using a Glutathione Fluorometric Assay Kit. Data are mean ± SD, *n* = 2. (**E**) The A375LM5*^IF4g/Luc^* cells (4 × 10^4^) were pre-treated with/without GSH (5 mM) for one hour and then incubated with vehicle, DET, or DETD-35 for 12 h and the migratory and invasive abilities were measured as described in Figure 3A. Top, cells stained with crystal violet. Bottom, crystal violet intensity of migrated/invaded cells detected at OD_595_. The scale bar represents 100 µm. Data are mean ± SD, *n* = 3. Means with significant differences are denoted with different letters (one-way ANOVA, *p* ≤ 0.05).

We examined whether the bioactivity of DET and DETD-35 against lung-seeking A375LM5*^IF4g/Luc^* melanoma cells was oxidative-stress dependent. First, we measured the intracellular ROS levels of A375LM5*^IF4g/Luc^* cells upon 1 h of DET or DETD-35 treatment using flow cytometry. As shown in Figure 4A, ROS levels of A375LM5*^IF4g/Luc^* increased from 100% in the vehicle-treated cells to 168% in DET- (6 µM) and 268% in DETD-35- (3 µM) treated cells. Glutathione (GSH) is a major intracellular non-protein thiol molecule that acts as a key antioxidant mechanism to maintain the redox homeostasis in mammalian cells [33]. Here, we found that exogenous supplementation with GSH at 5 mM reversed the DET/DETD-35-induced ROS levels to those similar to the vehicle-treated cells (Figure 4A). Moreover, GSH restored the A375LM5*^IF4g/Luc^* cell viability from 52% and 45% when cells were treated with DET and DETD-35, to around 100% (Figure 4B), and restored the DET- and DETD-35-induced apoptosis markers (cleaved PARP and cl. caspase 3) to levels comparable to the vehicle control (Figure 4C). Corresponding Western blotting quantification with bar graph is presented in Figure S1E. Of note, GSH did not affect the A375LM5*^IF4g/Luc^* cell viability or the expression of the apoptotic markers (Figure 4B,C). These data suggest that DET and DETD-35 treatment-initiated production of ROS might be through changes in thiol homeostasis, and that GSH might be important for the observed effects. We indeed observed that both compounds decreased the intracellular levels of GSH in A375LM5*^IF4g/Luc^* cells (Figure 4D), and that exogenously adding GSH in the culture medium can reverse the effect of both compounds. Collectively, the results indicate that both compounds induced cellular redox imbalance in part through the depletion of GSH and induction of ROS in the melanoma cells. DETD-35 exhibited a greater anti-lung-seeking melanoma cell effect, consistent with the higher production of ROS and lower GSH level.

ROS act as a double-edged sword in cancer progression and metastasis. Higher levels of ROS promote cancer cell metastasis; however, if the ROS levels exceed the anti-oxidative capacity of the cells, the metastatic abilities will be impaired [34]. Here, we found that when A375LM5*^IF4g/Luc^* were pre-treated with GSH for 1 h, the inhibition of migratory/invasive abilities by DET (6 µM) and DETD-35 (3 µM) treatment were reversed to the levels comparable to the vehicle control (Figure 4E). These results indicated that DET- and DETD-35-induced ROS generation in A375LM5*^IF4g/Luc^* melanoma cells might be an upstream factor important for the compounds’ anti-metastatic activities.

### 2.6. DET and DETD-35 Induced mtDNA Damage and Impaired Mitochondrial Function in A375LM5^IF4g/Luc^ Cells

**Figure 5:**
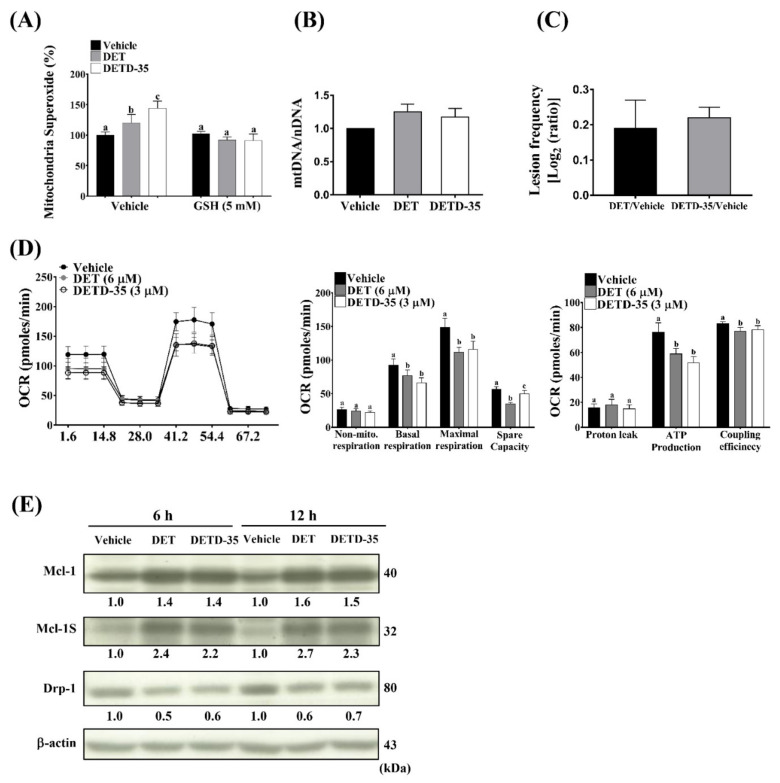
DET and DETD-35 induced mitochondrial dysfunction in A375LM5*^IF4g/Luc^* melanoma cells. (**A**) The A375LM5*^IF4g/Luc^* cells were pre-treated with/without GSH (5 mM) for one hour and then incubated with DET (6 µM) or DETD-35 (3 µM) for one hour in the presence of the mitochondria superoxide indicator MitoSox Red, and the levels of superoxide were detected by flow cytometry. (**B**,**C**) The A375LM5*^IF4g/Luc^* cells were treated with DET (6 µM) or DETD-35 (3 µM) for 6 h, the genomic DNA was isolated, and PCR products for nuclear and mitochondrial DNA were detected and quantified by agarose gel electrophoresis and ImageJ. The mitochondria mass was calculated by the formula mtDNA/nDNA, and the mtDNA lesion frequency was calculated by the formula: -LN((Large fragment/short fragment)/(average of large fragment/average of the control short fragment)). Data are mean ± SD, *n* = 3. (**D**) The A375LM5*^IF4g/Luc^* cells were treated with DET or DETD-35 for a total of 6 h, and the oxygen consumption rate (OCR) was measured using an XFp Cell Mito Stress Test Kit and Seahorse XFp Analyzer. Briefly, 6 h after treatment, OCR was measured under basal conditions and then after the sequential injection of mitochondria inhibitors. The inhibitors were oligomycin (1 µM), to decrease the electron flow through ETC; FCCP (1 µM), to promote maximum electron flow through ETC; and a mixture of rotenone (0.5 µM) and antimycin (0.5 µM), to shut down the mitochondria-related respiration. Data are mean ± SD, *n* = 4. Means with significant differences are denoted with different letters (one-way ANOVA, *p* ≤ 0.05). (**E**) A375LM5*^IF4g/Luc^* cells were incubated with vehicle, DET (6 µM) or DETD-35 (3 µM) for 6 h and 12 h. The protein expression levels were analyzed by Western blotting. Increased/decreased protein levels among treatments are presented as a fold change to the vehicle control after normalization to β-actin.

Mitochondria are one of the major sources of ROS in the cell, and are also the targets of ROS [16]. MitoSoxRed, superoxide indicator staining, and flow cytometry was applied to examine the levels of mitochondria-produced superoxide in the cells with or without the compound treatment. Figure 5A shows that superoxide levels were 100% in the vehicle control and elevated to 120% in DET- (6 µM) and 144% in DETD-35- (3 µM) treated cells. When pre-treated with ROS scavenger (GSH), the superoxide levels in the vehicle- or compound-treated cells were similar (Figure 5A). These data imply that ROS might be an upstream signal triggered by bioactive compounds to affect mitochondria function. MtDNA is more susceptible to mutation caused by oxidative stress than nuclear DNA (nDNA) due to the spatial proximity of the ROS species and the lack of nucleotide excision repair [35]. Here, we performed PCR-based analysis to measure the copy number of nDNA and mtDNA and to measure the mtDNA damage by quantifying the mutation-induced polymerase stalling lesions based on the PCR product amount [36]. We found that DET (6 µM) or DETD-35 (3 µM) treatment for 6 h induced mtDNA damage in A375LM5*^IF4g/Luc^* melanoma cells, as observed by increased lesion frequency ratios of DET and DETD-35 compared to the vehicle-treated control, which was referred to as mutation damage-free for the purpose of calculation (DET/Vehicle; DETD-35/Vehicle) (Figure 5C). We did not observe a significant difference in the mitochondrial copy number (mtDNA/nDNA) among treatment groups (Figure 5B), indicating that the observed mtDNA lesion frequency values were not due to changes in mitochondrial copy number upon DET or DETD-35 treatment. MtDNA mutations caused by ROS might impair the accurate synthesis of mtRNA templates for the mitochondrial electron transport chain protein complexes, which, on the other hand, might impact the electron transport-linked phosphorylation (OXPHOS) [37]. Given the observation that DET and DETD-35 caused mtDNA damage, we decided to examine the bioenergetic properties of A375LM5*^IF4g/Luc^* cells in response to DET and DETD-35.

The most useful test for the evaluation of mitochondrial function is measurement of the cell respiratory control. Therefore, we used a Seahourse XFp extracellular flux analyzer to test whether oxygen consumption rates (OCRs) were affected upon treatment with the compounds, allowing us to study OXPHOS in A375LM5*^IF4g/Luc^* cells. As shown in Figure 5D, 6 h of treatment with either DET (6 µM) or DETD-35 (3 µM) decreased the OCR. Furthermore, in the same experiment, by the sequential addition of oligomcycin (to decrease the electron flow through ETC), the uncoupling agent FCCP (to promote maximum electron flow through ETC), and a mixture of rotenone and antimycin (to shut down the mitochondria-related respiration), we were able to obtain abundant information about the basic parameters of cell respiratory control. DET and DETD-35 treatment resulted in a significant reduction in basal respiration, maximal respiration, and spare capacity of the A375LM5*^IF4g/Luc^*, cells indicating reduced mitochondrial capacity to produce energy under increased stress (Figure 5D). The ability of A375LM5*^IF4g/Luc^* cells to produce ATP was also reduced, along with reduced coupling capacity under both compound treatments (Figure 5D). The proton leak was slightly, but not significantly, increased by DET (Figure 5D). The non-mitochondrial respiration of A375LM5*^IF4g/Luc^* was not affected by either compound treatment (Figure 5D), indicating that DET/DETD-35 might not act by affecting enzymatic reactions and oxygenase outside the mitochondria. We further observed that DET/DETD-35 treatment for 6 h and 12 h induced increase in the full-length form of the anti-apoptotic protein Mcl-1; however, we also observed an increase in the Mcl-1 splicing variant Mcl-1S which antagonizes Mcl-1 and plays a pro-apoptotic role in cancer cells (Figure 5E) [38]. The mitochondria fission protein Drp1, which is important for the metastatic cell migratory activities [39], was found to be decreased upon 6 h and 12 h of DET or DETD-35 treatment (Figure 5E). Corresponding Western blotting quantification with bar graph is presented in Figure S1F. These results, taken together, suggest that impaired mitochondria function might be another mechanism that suppresses the metastatic abilities of A375LM5*^IF4g/Luc^* cells.

### 2.7. DET and DETD-35 Inhibited A375LM5^IF4g/Luc^ Lung Metastasis in Xenograft Mice

**Figure 6:**
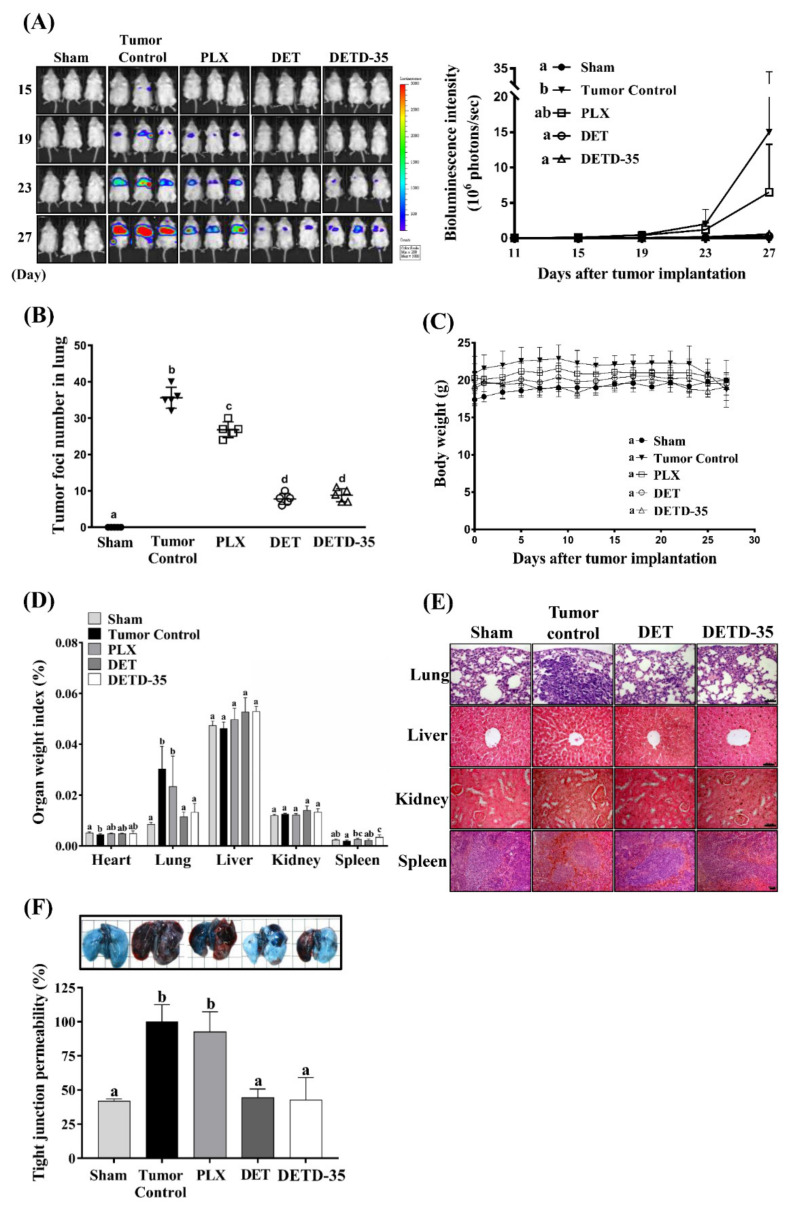
Effect of DET, DETD-35 and PLX on A375LM5*^IF4g/Luc^* lung metastasis in a xenograft mouse model. (**A**) Representative images of bioluminescence imaging (BLI) in the mice (left); the inhibitory effect of compounds on A375LM5*^IF4g/Luc^* lung metastasis in NOD/SCID mice shown by BLI between day 11 and day 27 (right). Data are mean ± SD, *n* = 5. (**B**) The metastatic melanoma nodules in paraffin-embedded lungs were counted, and the data are mean ± SD, *n* = 5. (**C**) The body weights of the test NOD/SCID mice were recorded during the experiment and are presented as mean ± SD, *n* = 5. (**D**) Organ weight index was calculated by dividing the organ weight to the body weight of the individual mice. Data are mean ± SD, *n* = 5. (**E**) The architecture and morphology of the lung, liver, kidney, and spleen were analyzed by H&E staining. (**F**) The pulmonary vasculature permeability was analyzed by Evans blue at the end of the study (day 27). Data are mean ± SD, *n* = 3. Means with significant differences are denoted with different letters (one-way ANOVA, *p* ≤ 0.05).

To evaluate the anti-metastatic effect of DET and DETD-35 in vivo, the lung-seeking A375LM5*^IF4g/Luc^* cells were injected into NOD/SCID mice and the melanoma metastasis into lungs was monitored by quantitative bioluminescence imaging (BLI). DET (20 mg/kg, intraperitoneal (i.p.), every other day) and DETD-35 (20 mg/kg, i.p., every other day), and the clinical BRAF inhibitor drug PLX (20 mg/kg, daily, i.p.) as a reference control, were used in this study. At the end of the study (day 27), the BLI analysis showed that compared to the tumor control (1.5 × 10^7^ photon/s), DET and DETD-35 treatment repressed the A375LM5*^IF4g/Luc^* melanoma lung metastasis in the mice by 98.1% (2.9 × 10^5^ photon/s) and 96.3% (5.6 × 10^5^ photon/s), respectively, and PLX treatment repressed the A375LM5*^IF4g/Luc^* melanoma lung metastasis in the mice by 42.4% (6.5 × 10^6^ photon/s) (Figure 6A). Furthermore, compared to the tumor control group, the number of melanoma foci in the paraffin-embedded lung tissues of the DET and DETD-35 treated groups was reduced by 78% and 75%, respectively, and had a superior effect in comparison with the PLX-treated group (24%) (Figure 6B). There was no significant body weight difference among the treatment groups (Figure 6C), and the organ index of major organs (liver, kidney, spleen, and lung) was not significantly different among the treatments, except that the spleen organ index in the mice of the DETD-35 treatment group showed statistical significance to other groups and that the mice in the PLX group showed increased lung organ index similar to the tumor control group due to the metastasis burden (Figure 6D). Histopathological examination of the hematoxylin and eosin (H&E) stained organs did not show any obvious difference in the liver, kidney, and spleen organ architecture among the treatment groups. The lung tissues revealed that the typical lung architecture with alveoli sacs as presented in the mouse lung tissues from the sham group was altered and filled with tumor mass in the lung tissues of the tumor control mice (Figure 6E). DET- and DETD-35-treated groups showed less alveolar sac damage and less tumor mass in the lungs, indicating that both treatments inhibited A375LM5*^IF4g/Luc^* melanoma metastasis and/or tumor growth in mouse lungs (Figure 6E).

During the pre-metastatic stage, cancer cells stimulate the permeability of the lung vasculature and prepare the lung microenvironment for colonization [40,41]. We used Evans blue (EB) leakage assay to examine the hyper-permeability of pulmonary capillaries elicited by the melanoma cells. The levels of EB were highest in the lungs of the tumor control mice, indicating the highest leakage of the lung vasculature (Figure 6F). The EB accumulated in the lungs of DET- and DETD-35-treated mice was significantly lower compared to the tumor control, suggesting that DET and DETD-35 inhibited tight junction permeability induced by metastatic A375LM5*^IF4g/Luc^* cells, and this might be one factor that contributed to their potent in vivo anti-metastatic effect (Figure 6F).

### 2.8. DET and DETD-35 Inhibited Various Biomarkers in the Lung Microenvironment of A375LM5^IF4g/Luc^ Xenograft Mice

**Figure 7:**
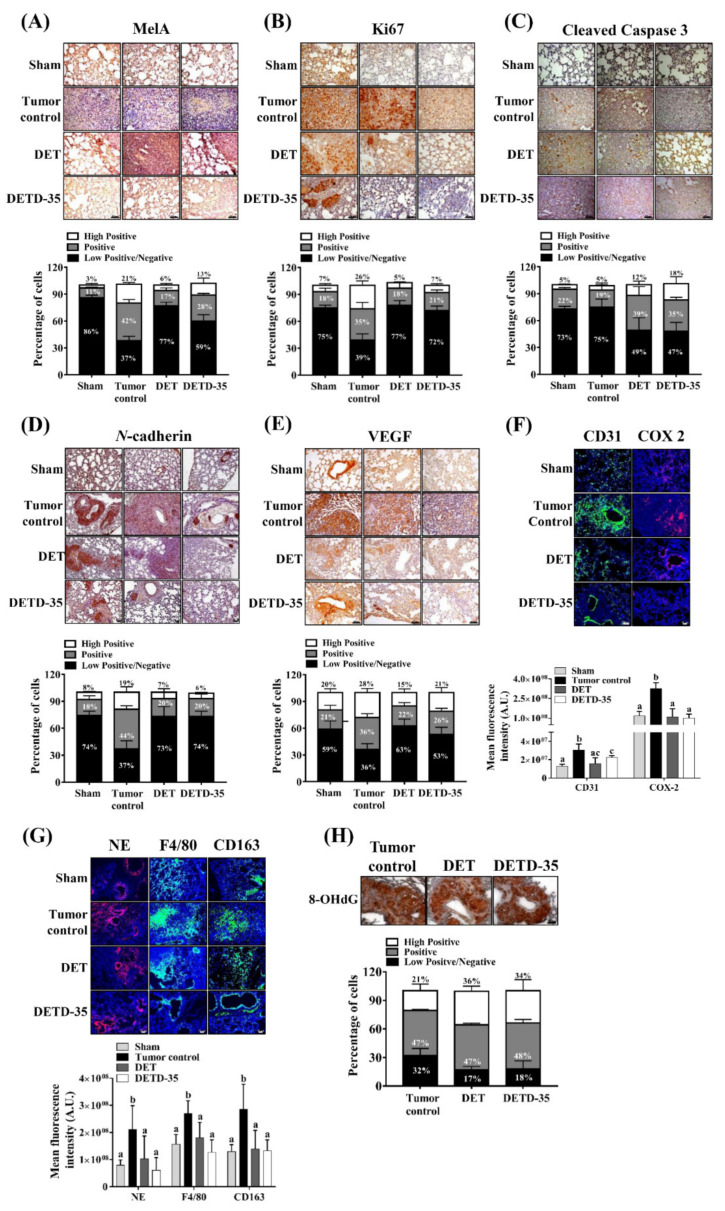
Effect of DET and DETD-35 on various protein markers in the lungs of metastatic melanoma mice. **A**–**E**: The representative images of (**A**) Mel-A, (**B**) Ki67, (**C**) cleaved caspase-3, (**D**) *N*-cadherin, and (**E**) VEGF expression in lung tissues from sham, tumor control, DET- (20 mg/kg) and DETD-35- (20 mg/kg) treated mice. The quantification data were analyzed by IHC profiler plugin, ImageJ. The percentage of positive-staining intensity for each specific protein categorized as high positive, positive, and low positive/negative are summarized. Data are mean ± SD, *n* = 4. **F**–**G**: The representative immunofluorescence images of (**F**) CD31 (green) and COX-2 (red), (**G**) Neutrophils (NE, red), macrophages (F4/80, green) and M2-like macrophages (CD163, green) in lung tissues from sham, tumor control, DET- (20 mg/kg) and DETD-35- (20 mg/kg) treated mice. The quantification was performed using ImageJ software. Data are mean ± SD, *n* = 4. Means with significant differences are denoted with different letters (one-way ANOVA, *p* ≤ 0.05). (**H**) The representative image of oxidative stress marker 8-OHdG in the tumor nodules of lungs from tumor control, DET- (20 mg/kg) and DETD-35- (20 mg/kg) treated mice. The quantification data were analyzed by IHC profiler plugin, ImageJ. The percentage of positive-staining intensity for 8-OHdG categorized as high positive, positive, and low positive/negative is summarized. Data are mean ± SD, *n* = 4. The scale bar represents 50 µm.

To further dissect the in vivo mechanism of melanoma growth and metastasis suppression, we examined the expression levels of proteins involved in melanoma proliferation, apoptosis, angiogenesis, and markers involved in melanoma progression and the inflammatory microenvironment in lung tissues from DET- or DETD-35-treated groups of mice, the tumor control group, and the sham group by immunohistochemistry (IHC) or immunofluorescence (IF). The IHC quantification was performed using ImageJ and IHC Profiler. The IHC images were assigned as high positive, positive, or low positive/negative when the percentage for a corresponding zone was equal to or higher than 33%. The IF quantification was performed by ImageJ and expressed as the mean fluorescence intensity. Based on these quantification methods, the melanoma marker Mel-A expression in the lung tissues was classified as positive and low positive/negative (42% and 37%) for the tumor control group, and as low positive/negative for the DET- (77%) and DETD-35-treated (59%) groups (Figure 7A), and the proliferation marker Ki67 was classified as positive and low positive/negative for the tumor control group (35% and 39%) and low positive/negative for the DET-treated (77%) and DETD-35-treated (72%) groups (Figure 7B). The cleavage form of caspase 3 was low positive/negative (75%) for the tumor control mice and positive and low positive/negative for the DET (39% and 49%) and DETD-35 (35% and 47%)-treated mice (Figure 7C). The expression level of *N*-cadherin was classified as positive and low positive/negative for the tumor control mice (36% and 36%), and low positive/negative for the DET (63%) and DETD-35 (53%) treatments (Figure 7D). The angiogenesis marker VEGF was positive and low positive/negative for the tumor control mice (44% and 37%), and low positive/negative for the DET-treated (73%) and DETD-35-treated (74%) mice (Figure 7E). The lung tissue of sham group mice was used as a reference for the normal expression of the examined proteins and for control of the color artifacts, and we found that all the markers in the lung tissue of the sham group mice were detected as low positive/negative (59–86%) (Figure 7A–E). The immunofluorescence quantification results revealed a significant decrease in the expression levels (fluorescence intensity) of neovascularization marker CD31 in the DET- and DETD-35-treated groups compared to the tumor control, supporting the above observation that both compounds block tumor angiogenesis in metastatic lung tissues (Figure 7F). Immune cells derived from bone marrow progenitor cells play a role in resolving inflammation; however, in the tumor microenvironment, the infiltrating monocytes, neutrophils, and tumor-associated macrophages support tumor angiogenesis and invasiveness [11]. The expression levels of monocyte/macrophage marker F4/80 and neutrophil marker NE in lung tissues from tumor control mice were higher than in the sham group, suggesting that there was tumor-elicited immune cell infiltration into the lung tissues (Figure 7G). Monocyte and neutrophil infiltration was prevented or reversed to a similar level to the sham group in the lung tissues from mice treated with DET or DETD-35. This was accompanied by a decrease in the presence of immune suppressive M2 macrophages in the lung tissues, as revealed by the significantly lower CD163 staining in DET- and DETD-35-treated mice compared to tumor control mice (Figure 7G). The expression level of COX-2, which might be produced by infiltrated immune cell or/and melanoma cells, was also significantly decreased by DET and DETD-35 (Figure 7F). Interestingly, we also observed that the expression of the marker for oxidative DNA damage 8-OHdG in the tumor nodules of the lungs was positive (47%) for the tumor control group, and high positive and positive for the DET- (36% and 47%) and DETD-35-treated (34% and 48%) groups, respectively (Figure 7H), in good agreement with the in vitro data that DET/DETD-35 induced oxidative stress in the lung-seeking melanoma cells that might result in DNA damage. All the results demonstrated that DET and DETD-35 impeded A375LM5*^IF4g/Luc^* metastatic melanoma growth by suppressing proliferation, angiogenesis, and EMT, and by modulating the lung microenvironment in mice.

## 3. Discussion

Melanoma is a cutaneous cancer with high metastatic potential. Despite the contemporary revolution in melanoma targeted and immune therapies, advanced melanoma patient survival remains poor. In this study, we created A375LM5*^IF4g/Luc^* BRAF-mutant lung-seeking melanoma cells and demonstrated that the phyto-sesquiterpene lactone DET and its semi-synthetic derivative DETD-35 effectively suppress metastasis in an A375LM5*^IF4g/Luc^* lung metastatic melanoma xenograft mouse model with bioefficacy that is superior to the clinically used BRAF inhibitor PLX. The underlying molecular mechanisms of both sesquiterpene lactones were mainly through the induction of oxidative stress-associated damage in metastatic melanoma cells.

ROS has a pivotal role in various cellular processes, including cancer cell proliferation, programed cell death, and metastasis. For example, mildly elevated ROS levels promote cell proliferation; however, in contrast, overproduction of ROS causes redox imbalance (oxidative stress), which leads to damage of the cell building blocks, loss of cell function, and apoptosis [17]. Many natural products promote cell apoptosis, which inhibits cancer growth and progression by inducing redox imbalance [42]. In this study, we demonstrated that oxidative stress is an important player in DET and DETD-35 anti-proliferative and apoptotic activities in A375LM5*^IF4g/Luc^* melanoma cells (Figure 4). In particular, we found that both compounds depleted intracellular levels of reduced GSH and increased the levels of intracellular ROS, which subsequently led to increased cytotoxicity and apoptosis. Furthermore, supplementation with GSH reversed the A375LM5*^IF4g/Luc^* melanoma cell ROS levels to the basal level and consequently reversed the anti-proliferative and apoptotic effects observed upon DET and DETD-35 treatment (Figure 4 and Figure 5). Both compounds induced increased generation of mitochondrial superoxide, which was also reversed by exogenous GSH supplementation, indicating that the increase in mitochondria superoxide is also a consequence of the redox imbalance in the cancer cells. From the Seahorse analysis (Figure 5), we observed that DET and DETD-35 affect mitochondria bioenergetics in A375LM5*^IF4g/Luc^* cells by inhibiting mitochondria oxidative phosphorylation, i.e., decreasing the basal oxygen consumption rate of A375LM5*^IF4g/Luc^* after compound treatment. Moreover, both compounds impaired the ability of the mitochondria to increase OXPHOS and to function at full capacity under cellular stressors (e.g., FCCP uncoupling agent treatment). We propose that the oxidative stress induced by DET and DETD-35 caused mtDNA damage or possibly mutation, which further impacts the production/function of proteins important for the electron transfer chain function, and consequently leads to the production of more ROS.

Most importantly, we demonstrated that intraperitoneal administration of DETD-35 or its parental compound sesquiterpene lactone DET inhibited A375LM5*^IF4g/Luc^* melanoma lung metastasis in a xenograft mouse model with no obvious toxicity to the mice and with better efficacy than PLX (Figure 6). The protein expression of *N*-cadherin, which is important for epithelial-to-mesenchymal transition in cells, was significantly decreased when A375LM5*^IF4g/Luc^* cells were treated with DET or DETD-35 (Figure 3C), which is consistent with the decrease in *N*-cadherin protein in the lung tissues of mice treated with DET or DETD-35 (Figure 7D). The decreased level of *N*-cadherin might be important for A375LM5*^IF4g/Luc^* cell reprograming prior to colonizing the lung tissue, and thus the anti-metastatic effect of the compounds. COX-2 is a valuable prognostic marker for metastatic melanoma, and strong staining correlates with high Clark levels and poor outcome (involvement of lymph nodes and metastasis) [43,44]. Epidemiological studies have shown that COX-2 inhibitors (e.g., aspirin) reduced the incidence of several cancers. In this study, we found decreased expression of COX-2 in metastatic lung tissues of DET- and DETD-35-treated mice compared to tumor control mice (Figure 7F). In previous studies from our lab, COX-2 overexpression in the lungs with TS/A and MD-MBA-231 metastasis was significantly repressed by DET and DETD-35, respectively [22,25]. We speculate that the profound inhibition of A375LM5*^IF4g/Luc^* lung metastasis by both compounds is partly through suppressing COX-2 overexpression, which was the case with other natural product or inhibitor drugs [45,46,47].

The lung is one of the most common melanoma metastasis organs due to its dense vasculature. Here, we found that DET and DETD-35 decreased the permeability of the pulmonary vasculature as detected by the tight junction permeability assay; therefore, possibly causing the decrease in melanoma cell infiltration into the lungs (Figure 6F). Furthermore, it also inhibited angiogenesis because the expression of angiogenic marker VEGF and endothelial marker CD31 were attenuated by both compound treatments (Figure 7E,F). On the other hand, high infiltration of macrophages into the tumor microenvironment (TME) is linked to poor prognosis in several cancers including melanoma. Tumor-associated macrophages have a tumor-promoting role through governing tumor cell proliferation, tumor angiogenesis, invasion, and metastasis [48]. In this study, we found a decreased presence of macrophages (positive F4/80 staining) in the lung tissues of mice treated with DET or DETD-35 accompanied by the decreased presence of immunosuppressive macrophages (positive CD163 staining). These data imply that both compounds may inhibit TME-driven M2 macrophage polarization. Furthermore, there was a decreased presence of neutrophils (positive NE staining) into the lungs of DET and DETD-35 mice compared to the tumor control (Figure 7G). However, whether the decreased presence of macrophages or neutrophils is the result of TME modulation by DET or DETD-35, decreased pulmonary vascular permeability, or simply by impairing the ability of monocytes/neutrophils to migrate into the lungs warrants further study.

Accumulating knowledge including several structure–activity relationship studies on plant sesquiterpene lactones demonstrated that the presence of an alkylating center (α-methylene-γ-lactone, α-methylene-δ-lactone, conjugated cyclopentenone or conjugated side chain ester) is essential for their anti-cancer and immunomodulatory activity, and that sesquiterpene lactone-induced ROS generation or impaired detoxification might be an upstream regulator of those activities [20,49]. For example, the endoperoxide bridge in artemisinin was shown to react with endogenous ions in cancer cells and to cause the generation of toxic free radicals, and subsequently, cancer cell death [49]. The reaction of the lactone moiety of parthenolide with the intracellular reduced glutathione was also shown to disturb the redox homeostasis and to induce toxic ROS in cancer cells [50]. However, whether DET- and DETD-35-induced GSH depletion and ROS elevation is due to direct interaction of the compounds’ lactone moiety to the GSH or due to the dysregulation of GSH synthesis warrants further study. Although we observed that DETD-35 at the same or lower concentration to DET was more potent in inhibiting A375LM5*^IF4g/Luc^* activities in vitro, which might be due to the increased lipophilicity and increased penetration into the cell due to the presence of the naphthalene ring, the in vivo anti-metastatic effect of DETD-35 at the same administration dose (20 mg/kg) was comparable to DET (20 mg/kg) (Figure 6A,B). One important factor contributing to this result might be that at the same compound weight, the molar quantity of DETD-35 was lower than that of DET due to the higher molecular weight (444.5 vs. 344.3). Whether this effect is also as a result of the absorption, distribution, metabolism and excretion (ADME) properties of the compounds, or whether modification of the dose and administration route or delivery method will potentiate DETD-35 anti-melanoma in vivo activity, needs further investigation.

## 4. Materials and Methods

### 4.1. Materials

The 3-(4,5-Dimethylthiazol-2-yl)-2,5-diphenyltetrazolium bromide (MTT), dimethyl sulfoxide (DMSO), crystal violet, and reduced glutathione were purchased from Sigma-Aldrich (St. Louis, MO, USA). Dulbecco’s Modified Eagle Medium (DMEM), fetal bovine serum (FBS), and antibiotic mixture (penicillin–streptomycin) were from Invitrogen (Carlsbad, CA, USA). Vemurafenib (PLX4032, designated PLX in this study) was purchased from Selleck Chemical (Houston, TX, USA), dissolved in DMSO as a stock solution of 25 µM, and stored at −20 °C. The working concentration for the in vitro assays was 10 µM.

### 4.2. Compounds DET and DETD-35

The source compound DET was isolated and purified from the medicinal plant *Elephantopus scaber* (Asteraceae) following a method published elsewhere [22]. DETD-35 was synthesized from DET also following a previously published method [25]. Both compounds were dissolved in DMSO as a stock solution of 50 µM stored at −20 °C. The working concentration for the in vitro assays was 10 µM for both compounds.

### 4.3. Cell lines and Culture Conditions

The A375 human malignant melanoma cell line (BRAF*^V600E^* mutant) was obtained from ATCC (Manassas, VA, USA) and grown in DMEM containing 10% fetal bovine serum, 100 units/mL penicillin, and 100 μg/mL streptomycin at 37 °C in a humidified 5% CO_2_ incubator. A375LM5*^IF4g/Luc^*, a lung-seeking metastatic melanoma cell line, carrying hEF1α-elF4g promoter-driven luciferase reporter gene was established in-house by five repeated cycles of in vivo/ex vivo primary culture. The A375LM5*^IF4g/Luc^* cells were cultured under the same conditions as the A375 cells, but the culture media was supplemented with blasticidin (1:1500).

### 4.4. Cell Viability Assay

A375 and A375LM*^IF4g/Luc^* cells were seeded in 96-well plates for 12 h, and then the indicated concentrations of the tested compounds were added to the culture media which was incubated for 24 h or 72 h. Cell viability of A375 and A375LM*^IF4g/Luc^* melanoma cells was examined by MTT assay and calculated as follows: viable cells (%) = [OD_570_ (compound treated cells)/OD_570_ (vehicle treated cells)] × 100.

### 4.5. Colony Formation Assay

The colony-forming ability of A375LM5*^IF4g/Luc^* melanoma cells was analyzed by seeding 1 × 10^3^ A375LM5*^IF4g/Luc^* cells in 24-well plates for 12 h. The cells were incubated with the indicated concentrations of DET, DETD-35 or PLX for six days. The culture medium was replaced on the third day after seeding with the same treatments. At day 6, cell colonies were first fixed with 250 µL of ice-cold methanol and then stained with 250 µL of 0.1% (*w*/*v*) crystal violet solution for 30 min. After air-drying, the cell colonies were stained with crystal violet and dissolved in 250 µL of 20% (*v*/*v*) acetic acid for 15 min and measured by absorbance at 595 nm.

### 4.6. Cell Cycle Analysis

The A375LM5*^IF4g/Luc^* cells were synchronized by incubation in 5% FBS DMEM culture medium for 8 h first, and then incubated in 0.5% FBS DMEM for 24 h. The synchronized cells were incubated with/without DET or DETD-35 with 10% FBS DMEM for 24 h. The DNA distribution in different treatments was detected as described previously [51].

### 4.7. Apoptosis Assay

The A375LM5*^IF4g/Luc^* (1.5 × 10^5^/well) were seeded in 6-well plates, and 12 h later were treated with/without DET or DETD-35 for 48 h. Apoptotic populations were measured using the FITC-Annexin V Apoptosis Detection Kit following the manufacturer’s suggestions (BD Pharmingen, San Diego, CA, USA) and BD LSR II cytometer (BD LSR II, BD Biosciences, San Jose, CA, USA).

### 4.8. Western Blot Analysis

Total cellular proteins were prepared according to our lab’s protocol [51]. Tested samples were resolved by SDS-PAGE (10% or 12%) and then underwent immunoblotting. Primary antibodies against cdc2 p34 (sc-54, 1:1000), p-cdc2 p34 (sc-12341, 1:1000), cyclin B1 (sc-594, 1:1000), Cdc25C (sc-327, 1:1000), caspase 3 (sc-56053, 1:500), PARP (sc-7150, 1:2000), Mcl-1 (sc-819, 1:500), and DRP1 (sc-101270, 1:500) were purchased from Santa Cruz Biotechnology (Dallas, TX, USA), Vimentin (10336-1-AP, 1:2000), MMP-2 (10373-2-AP, 1:1000), *N*-cadherin (22018-1-AP, 1:2000), *E*-cadherin (20874-1-AP, 1:1000), α-SMA (55135-1-AP, 1:1000), and integrin alpha-4 (19676-1-AP, 1:1000) were purchased from Proteintech (Taipei, Taiwan), and β-actin (MAB1501, 1:10,000) was from Merck Millipore (Burlington, MA, USA).

### 4.9. Cell Migration/Invasion Assay

Migration ability of the A375LM5*^IF4g/Luc^* cells in the presence or absence of DET or DETD-35 was measured using 24-well Boyden chamber cell culture inserts (8.0 µm pore polycarbonate filter) (Cat. PIEP12R48, Millipore). The A375 or A375LM5*^IF4g/Luc^* cells resuspended in 100 µL 1% FBS-containing DMEM media were seeded onto the apical side of the inserts. One milliliter of 10% FBS-containing media (as a chemoattractant) in the presence or absence of the indicated compounds was supplemented in the lower chamber. Where indicated, cells were pre-treated for 1 h with 5 mM of GSH in 1% FBS-containing media, and the media was further replaced with fresh media containing the indicated compounds. Twelve hours after the compound treatments, the cells on the apical side of the insert were removed by wiping with cotton swaps, and the migrated cells on the basal side of the insert were fixed with 0.5 mL of ice-cold methanol (15 min), stained with 0.5 mL 0.1% crystal violet (15 min), rinsed with 0.5 mL PBS, and photographed on an inverted microscope (Zeiss AXiovert 200M). The invasion assay was performed under the same procedure, except that prior to cell seeding, the apical side of the inserts was coated with 300 µg/mL Matrigel (Cat. #356237, BD Bioscience) dissolved in 1% FBS-containing DMEM culture media. The quantification of the migrated or invaded cells for Figure 1C was performed by counting the number of migrated or invaded A375/A375LM5 cells. The number of migrated/invaded A375 or A375LM5 cells was first normalized to the number of seeded cells A375 (1 × 10^5^ cells) or A375LM5*^IF4g/Luc^* (3 × 10^4^ cells) and further expressed as the migration or invasion percentage to the parental cells, where parental cell migration or invasion was designated as 100%. The quantification of migrated or invaded cells for Figure 3A and Figure 4E was performed when cell-retaining crystal violet was dissolved in 250 µL 20% acetic acid and the absorbance was measured at 595 nm using an Elisa reader. Migrated/invaded cells were calculated as follows: Migrated/Invaded cells (%) = OD_595_ (compound treated cells)/OD_595_ (vehicle treated cells) × 100.

### 4.10. ROS Measurement

The intracellular ROS levels in A375LM5*^IF4g/Luc^* cells were measured using the Total ROS Detection Kit (Cat. #51011, Enzo Life Sciences, Farmingdale, NY, USA). The A375LM5*^IF4g/Luc^* cells (1.5 × 10^5^) were seeded into 6-well plates, and 12 h later were incubated for one hour in 3 mL of phenol red-free DMEM culture media containing the indicated concentration of DET or DETD-35 and 1 µM of Oxidative Stress Detection Reagent (green probe), which reacts directly with hydrogen peroxide, hydroxyl radical, nitric oxide, peroxyradicals, and other reactive species to generate a green, fluorescent signal. Where indicated, A375LM5*^IF4g/Luc^* cells were incubated with 3 mL of culture media containing 5 mM GSH one hour prior to compound treatments. After the treatment cells were trypsinized, washed with PBS, and resuspended in PBS, the generated ROS levels were measured by flow cytometry (BD LSR II, BD Lifesciences, San Jose, CA, USAThe same approach was used for the detection of mitochondria-generated superoxide, except that the cells were incubated with 2.5 µM of mitochondria superoxide indicator MitoSOX Red reagent (M36008, Molecular Probes, Invitrogen, Waltham, MA, USA).

### 4.11. GSH Measurement

The content of glutathione (GSH) was detected using the Glutathione Fluorometric Assay Kit (K264, BioVision, Milpitas, CA, USA). Briefly, the A375LM5*^IF4g/Luc^* cells (2 × 10^6^) were seeded into a 15 cm dish, and 12 h later were treated with/without DET or DETD-35. One hour after the treatment, cells were collected, resuspended in 100 µL ice-cold GSH Assay Buffer, and then 60 µL of the cell homogenate was added to 20 µL of ice-cold PCA and vortexed for a few seconds. Five minutes after incubation on ice, the emulsion was centrifuged (13,000× *g*, 2 min, 4 °C) and the supernatant containing GSH was collected. PCA preserved samples (40 µL) were neutralized by the addition of 20 µL ice-cold 6N KOH, and after 5 min of incubation on ice, the samples were centrifuged (13,000× *g*, 2 min, 4 °C) and the supernatant was collected for analysis. The supernatant (10 µL) was loaded on to 96-well plate, mixed with 80 µL of Assay Buffer and 10 µL OPA probe; 40 min after incubation in the dark, the GSH levels (fluorescence intensity) were read by a florescence plate reader at 340/420 nm. The GSH concentration was calculated as follows: GSH concentration = GSH amount from standard curve (µg)/sample volume added to the sample wells (ml).

### 4.12. Mitochondrial DNA Damage Assay

A375LM5*^IF4g/Luc^* cells were seeded overnight in 10 cm culture dishes and then treated with vehicle, DET, or DETD-35 for 6 h. After compound treatment, total genomic DNA was extracted according to the manufacturer’s instructions. PCR was performed using the following primers: 5′-CGA AAG CAT TTG CCA AGA AT-3′ and 5′-AGT CGG CAT CGT TTA TGG TC-3′ for the nuclear DNA fragment; 5′-CCC CAC AAA CCC CAT TAC TAA ACC CA-3′ and 5′-TTT CAT CAT GCG GAG ATG TTG GAT GG-3′ for the short 221 bp mitochondria fragment; and 5′-TCT AAG CCT CCT TAT TCG AGC CGA-3′ and 5′-TTT CAT CAT GCG GAG ATG TTG GAT GG-3′ for the long 8.9 kb mitochondrial fragment. The reaction conditions for the amplification of the nuclear DNA and the mitochondrial small fragment were as follows: denaturation at 94 °C for 15 s; 30 cycles of 15 s at 94 °C, 10 min at 60 °C; and extension for 1 min at 60 °C. The mitochondria large fragment was amplified under the following conditions: denaturation for 10 s at 98 °C; 35 cycles of 10 s at 98 °C, 30 s at 64 °C and 6 min at 72 °C; and extension for 10 min at 72 °C. PCR products were resolved on 1% agarose gel and quantified using ImageJ.

### 4.13. Cell Mitochondria Stress Test

Mitochondrial respiratory function was examined using Seahorse XFp Cell Mito Stress Test Kit (Cat. #103010-100, Agilent Technologies, Santa Clara, CA, USA) according to the manufacturer’s instructions. Briefly, 1.75 × 10^4^ A375LM5*^IF4g/Luc^* cells per well resuspended in 80 µL regular DMEM media were seeded in an XFp miniplate. Twelve hours later, the media was removed, the wells were washed once with 180 µL of Seahorse XF Base Media (Cat.# 103335-100) supplemented with 2% FBS, 4 mM glutamine, 25 mM glucose, and further refreshed and incubated with 180 µL of the same media containing vehicle, 6 µM DET, or 3 µM DETD-35 in a non-CO_2_ incubator at 37 °C. Four and half hours after the incubation, the cell culture XFp miniplate was loaded into the Seahorse XFp analyzer (Seahorse Biosciences, Agilent Technologies, Santa Clara, CA, USA). Real-time oxygen consumption rate was measured at baseline (Basal OCR) for 1.5 h prior to mitochondrial perturbation by sequential injection of 1 µM oligomycin (a complex V inhibitor to decrease the electron flow through ETC); 1 µM FCCP (the uncoupling agent to promote maximum electron flow through ETC); and a mixture of 0.5 µM rotenone and 0.5 µM antimycin (complex I and complex II inhibitors, respectively, to shut down the mitochondria-related respiration). The data were analyzed using Seahorse Wave Desktop Software (Agilent Technologies) and the parameter values were calculated using Seahorse XF Cell Mito Stress Test Parameter Equations (Seahorse Report Generator User Guide, Agilent Seahorse XF Cell Mito Stress Test, Agilent Technologies, Santa Clara, CA, USA).

### 4.14. Animals

The animal study was performed according to a protocol approved by the Institutional Animal Care and Utilization Committee (IACUC) of Academia Sinica, Taipei, Taiwan. The NOD/SCID (NOD.CB17-*Prkdc*^scid^/NcrCrl) female mice were bred in the Laboratory Animal Core Facility at the Agricultural Biotechnology Research Center (ABRC-LACF), Academia Sinica. Mice were given a distilled water and standard laboratory diet ad libitum and kept in a 12 h light/dark cycle at 22 ± 2 °C and 55 ± 5% humidity.

### 4.15. Experimental Lung Metastasis Mouse Model

At day 0, the A375LM5*^IF4g/Luc^* cells (3 × 10^5^) resuspended in PBS (200 mL) were intravenously (i.v.) injected into the tail vein of the NOD/SCID mice (female, 4 weeks old) and randomized into five groups (*n* = 8 in each group), except for the sham group which were injected with PBS only and did not receive any further operation. Starting from day 1, animals were treated with vehicle (5% DMSO and 1% Tween 80 in 0.2 mL of PBS, i.p.; Tumor control), DET (20 mg/kg body weight, i.p.; DET), and DETD-35 (20 mg/kg body weight, i.p.; DETD-35) every second day, or PLX (20 mg/kg body weight, i.p.; PLX) once daily. Mice were weighed every two days from the beginning of the treatment, and in vivo bioluminescence was measured every two days from day 13 until the endpoint of the study. Briefly, animals were anesthetized with 2.5% isoflurane/air mixture, intraperitoneally administered with 150 mg/kg/BW D-luciferin (D-luciferin potassium salt, Gold Biotechnologies, St. Louis, MO, USA), and 10–15 min later when the luciferase signal reached a plateau, the photon emission from the luciferase activity in mice was acquired by the IVIS spectrum system (Xenogen Corp., Hopkinton, MA, USA). Quantification of the signal intensity was performed by Living Image 2.5 (Xenogen Corp.). At day 27, the mice were sacrificed, and the organs (liver, kidney, spleen, and lung) were prepared for histopathological analysis.

### 4.16. Tight Junction Permeability Analysis

The tight junction permeability assay was performed as described previously [52]. Briefly, on the 27th day of the animal study, the five experimental groups (sham, tumor control, PLX, DET, and DETD-35, *n* = 3) of A375LM*^IF4g/Luc^* lung metastatic melanoma mice were injected (i.v.) with 160 mg/kg/mouse Evans blue (E2129, Sigma) solution. Mice were perfused with 0.9% saline solution, mouse lungs were excised, and the EB dye retained in the lung tissues was extracted by overnight incubation of the lungs in formamide solution (Cat. 4028-01, J.T.Baker) at 37 °C. Furthermore, the formamide solution containing EB was collected after centrifugation (13,000× *g*, 30 min) and the concentration was calculated after detection of the absorbance (OD_590_).

### 4.17. Immunofluorescence and Immunohistochemistry of Lung Tissues from A375LM5^IF4g/Luc^ Metastatic Mouse Model

For immunofluorescence staining of lung tissues from the A375LM5*^IF4g/Luc^* metastatic melanoma mouse model, the primary antibody against Neutrophil elastase (ab68672, Abcam, 1:200), CD31 (11265-1-AP, Proteintech, 1:200), CD163 (16646-1-AP, 1:200), F4/80 (cat. #123101, Biolegend, 1:200), COX-2 (sc-19999, Santa Cruz, 1:200) were probed with Alexa Fluor 488 or Alexa Fluor 594 conjugated secondary antibody (1:1000 dilution) (Bio-Rad Laboratories, Hercules, California, USA). The quantification of the protein expression (antibody fluorescence intensity) in the lung tissue was performed using ImageJ software. The primary antibodies Mel-A (ab186731, 1:200) Ki67 (ab8191, 1:200) and 8-OHdG (ab48508, 1:400) from Abcam (Cambridge, UK), anti-Caspase 3 cleaved form (AB3623, 1:200) from Merek (Darmstadt, Germany) and VEGF (19003-1-AP, 1:200) and *N*-cadherin (22018-1-AP, 1:200) from Proteintech (Taipei, Taiwan) were used for immunohistochemistry. The quantification of the protein expression (antibody DAB staining) in the lung tissue was performed using ImageJ and plug-in IHC Profiler [53]. This tool creates pixel-by-pixel analysis of the DAB-stained tissue image and further performs automated scoring in a three-tier system as high positive, positive, and low positive/negative according to the color intensity with respect to the darkest DAB-stained image area and the unstained image background. The IHC images from the examined tissues were assigned as high positive, positive, or low positive/negative when the percentage for a corresponding zone was equal to or higher than 33%.

### 4.18. Statistical Analysis

Quantification of all experimental data are represented as mean ± standard deviation (SD) with the number of experiments indicated in the figure legends. Statistical analysis was conducted by Predictive Analysis Suite Workstation (PASW Statistics 18, Chicago, IL, USA) and significant differences within treatments were determined by one-way ANOVA. *p* ≤ 0.05 was considered as statistically significant.

## 5. Conclusions

In this study, we observed that, in vitro, DET and DETD-35 act through the induction of ROS-mediated apoptosis and downregulation of metastasis markers, inhibition of migration/invasion, and by impairing the mitochondrial function in lung-seeking A375LM5*^IF4g/Luc^* melanoma cells. The in vitro bioactivity results are in good agreement with the in vivo results observed in the A375LM5*^IF4g/Luc^* melanoma lung metastasis xenograft mouse model, i.e., showing that DET or DETD-35 deregulated tumor cell proliferation, apoptosis, and metastasis-related protein expression, and affect the lung microenvironment dynamics and lung capillary permeability. This work, therefore, further validates the potential of phytosesquiterpene lactone DET and its derivative in the treatment of advanced melanoma.

## Data Availability

All of the data is presented in the article and supplementary file. No additional data is reported.

## Supplementary Materials

The following are available online at https://www.mdpi.com/1422-0067/22/6/3226/s1.

## Abbreviations

A375LM5*^IF4g/Luc^*: lung metastatic BRAF mutant human A375 melanoma cell line with stable firefly luciferase reporter gene driven by a hybrid EF1α/eIF4g promoter. BRAF: V-raf murine sarcoma viral oncogene homolog B1, BRAF^V600E^: activating point mutation with substitution of valine to glutamic acid at residue 600 of BRAF, DET: deoxyelephantopin, DETD-35: DET semi-synthetic derivative-35, PLX: PLX4028.
