# Supplementary material for: Sesquiterpene Lactone Deoxyelephantopin Isolated from Elephantopus scaber and Its Derivative DETD-35 Suppress BRAFV600E Mutant Melanoma Lung Metastasis in Mice
Source: Int J Mol Sci. 2021 Mar 22;22(6):3226. doi: 10.3390/ijms22063226 (PMC8004649; doi:10.3390/ijms22063226)
Supplement: Supplementary file 1 [file ijms-22-03226-s001.pdf]

Supplementary Information

Supplementary Figure S1. Bar graphs for the western blot data form the main Figure 1B, Figure 2C, Figure 2E, Figure 3C, Figure 4C, and Figure 5E.

(A) Bar graph corresponding to Figure 1B. Western blotting was performed to check the expression of the basal levels of metastasis-related proteins in A375, A375LM$^{IF4g/Luc}$, A375LM3$^{IF4g/Luc}$, and A375LM5$^{IF4g/Luc}$ melanoma cells. Increased/decreased protein levels among different cells are presented as a fold change to the vehicle control after normalization to β-actin.

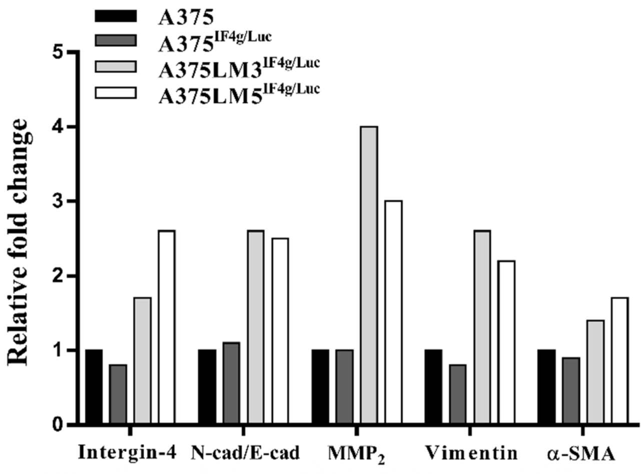

(B) Bar graphs corresponding to Figure 2C. The melanoma ling-seeking A375LM5$^{IF4g/Luc}$ cells were incubated with vehicle, DET, DETD-35 or PLX for 24 h and the expression of the cell cycle proteins were analyzed using western blotting. Increased/decreased protein levels among treatments are presented as a fold change to the vehicle control after normalization to β-actin.

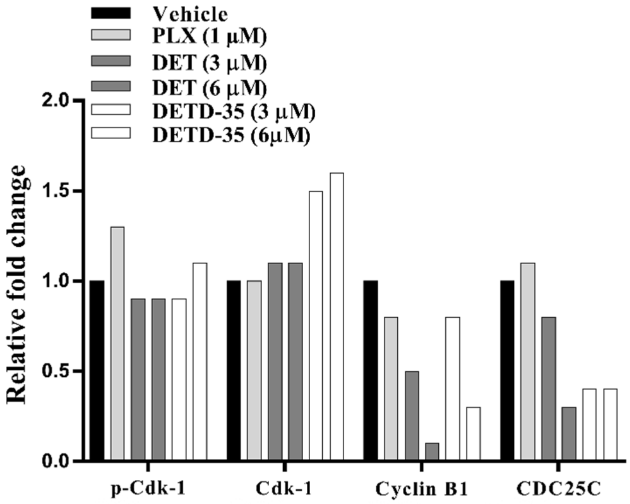

(C) Bar graphs corresponding to Figure 2E. The melanoma lung-seeking A375LM5$^{IF4g/Luc}$ cells were incubated with vehicle, DET, DETD-35 or PLX for 48 h and the expression of the apoptotic proteins were analyzed by western blotting. Increased/decreased protein levels among treatments are presented as a fold change to the vehicle control after normalization to β-actin.

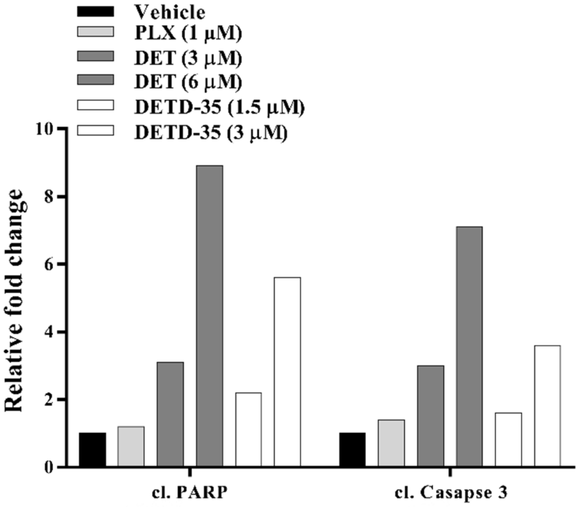

(D) Bar graphs corresponding to Figure 3C. A375LM5$^{IF4g/Luc}$ cells were incubated with vehicle, DET, DETD-35 and PLX for 48 h. The expression levels of metastatic protein markers were analyzed by western blotting. Increased/decreased protein levels among treatments are presented as a fold change to the vehicle control after normalization to β-actin.

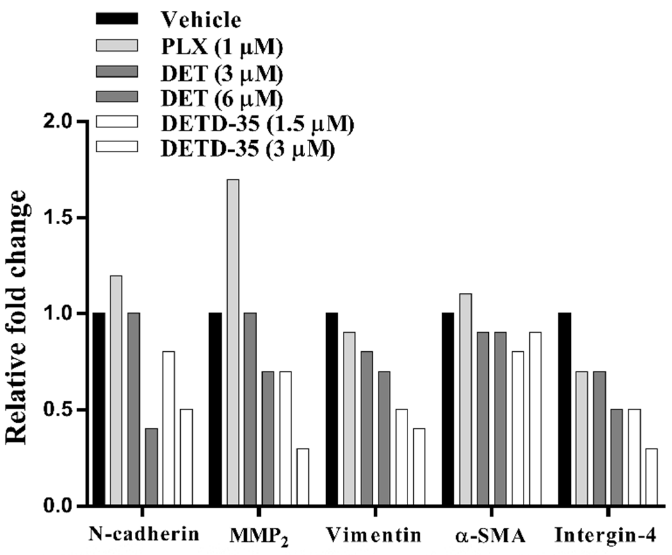

(E) Bar graphs corresponding to Figure 4C. The melanoma lung-seeking A375LM5$^{IF4g/Luc}$ cells were pre-treated with/without GSH (5 mM) for one hour and then incubated with vehicle, DET or DETD-35 for 48 h and expression levels of apoptotic protein markers were analyzed by western blotting. Increased/decreased protein levels among treatments are presented as a fold change to the vehicle control after normalization to β-actin.

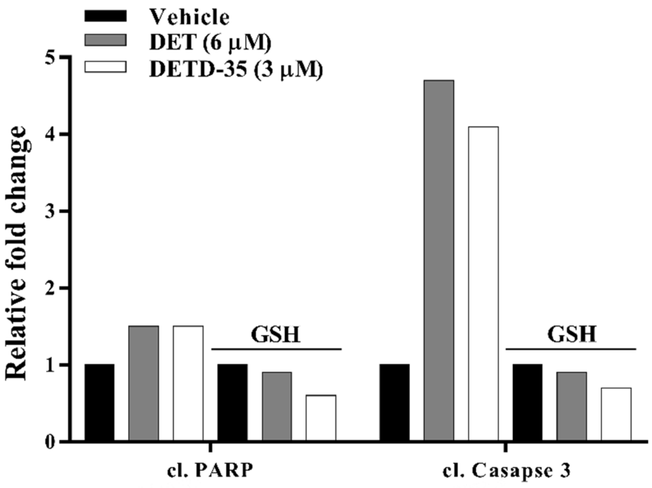

(F) Bar graphs corresponding to Figure 5E. (E) A375LM5$^{IF4g/Luc}$ cells were incubated with vehicle, DET or DETD-35 for 6 h and 12 h. The protein expression levels were analyzed by western blotting. Increased/decreased protein levels among treatments are presented as a fold change to the vehicle control after normalization to β-actin.

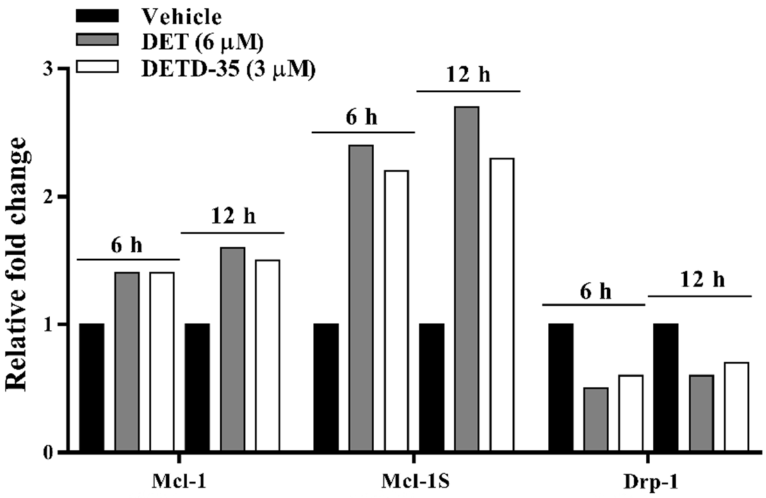
Vehicle
DET (6 μM)
DETD-35 (3 μM)
Relative fold change
3
2
1
0
6 h
12 h
6 h
12 h
6 h
12 h
Mcl-1
Mcl-1S
Drp-1
